# Structure and Compressive Properties of Invar-Cenosphere Syntactic Foams

**DOI:** 10.3390/ma9020115

**Published:** 2016-02-18

**Authors:** Dung Luong, Dirk Lehmhus, Nikhil Gupta, Joerg Weise, Mohamed Bayoumi

**Affiliations:** 1 Composite Materials and Mechanics Laboratory, Mechanical and Aerospace Engineering Department, Tandon School of Engineering, New York University, Brooklyn, NY 11201, USA; ngupta@nyu.edu; 2 ISIS Sensorial Materials Scientific Centre, University of Bremen, Bremen D-28359, Germany; dirk.lehmhus@uni-bremen.de; 3 MAPEX Center for Materials and Processes, University of Bremen, Bremen D-28359, Germany; 4 Fraunhofer Institute for Manufacturing Technology and Advanced Materials (IFAM), Bremen D-28359, Germany; joerg.weise@ifam.fraunhofer.de; 5 Department of Mechanical Engineering, Al-Azhar University, Cairo 11651, Egypt; m-bayoumi@azhar.edu.eg

**Keywords:** syntactic foams, metal foams, metal matrix composites, high strain rate, strain rate sensitivity, split-Hopkinson pressure bar

## Abstract

The present study investigates the mechanical performance of syntactic foams produced by means of the metal powder injection molding process having an Invar (FeNi36) matrix and including cenospheres as hollow particles at weight fractions (wt.%) of 5 and 10, respectively, corresponding to approximately 41.6 and 60.0 vol.% in relation to the metal content and at 0.6 g/cm^3^ hollow particle density. The synthesis process results in survival of cenospheres and provides low density syntactic foams. The microstructure of the materials is investigated as well as the mechanical performance under quasi-static and high strain rate compressive loads. The compressive stress-strain curves of syntactic foams reveal a continuous strain hardening behavior in the plastic region, followed by a densification region. The results reveal a strain rate sensitivity in cenosphere-based Invar matrix syntactic foams. Differences in properties between cenosphere- and glass microsphere-based materials are discussed in relation to the findings of microstructural investigations. Cenospheres present a viable choice as filler material in iron-based syntactic foams due to their higher thermal stability compared to glass microspheres.

## 1. Introduction

Syntactic foams (SFs) are foams in which porosity is introduced via hollow particles dispersed within a matrix material. Polymer matrix SFs have found widespread application in the offshore and oil industry, where they serve as buoyancy devices that can withstand much higher hydrostatic loads than other types of foams [1,2]. Such superior mechanical performance is attributed to the considerable strength of hollow particles used not only as lightweight filler, but also in the strengthening phase. Prominent among these filler materials are hollow glass microspheres, fly ash cenospheres, and ceramic and in some cases even metallic hollow spheres [3,4]. Of these, the former two are available in size ranges below a few hundred micrometers, while the ceramic spheres typically come in sizes ranging from micrometers to millimeters.

Metal matrix SFs (MMSFs) also show great application potential, based both on performance and cost, but their adoption in industry has been slow [5,6,7]. Processes for their production are typically either melt-based or powder metallurgical techniques. Melt infiltration of either geometrically confined loose hollow particles or of sintered or otherwise bonded preforms has been demonstrated for several matrix materials: aluminum matrix syntactic foams have, for example, been produced in combination with various fillers by Weise *et al.* (glass microspheres, [8]), Luong *et al.* (cenospheres, [9]) or Szlancsik *et al.* (Fe hollow spheres, [10]) while magnesium matrices have been scrutinized by Gupta *et al.* [11] and Luong *et al.* [12]. Zinc foams have also been synthesized using pressure assisted infiltration [8]. Higher melting matrix materials are typically processed powder metallurgically: there are exceptions in the case of iron and steel matrices [13,14], but titanium, for example, is generally treated using powder metallurgy (PM) techniques due to its even higher melting point and reactivity [15,16].

**Figure 1:**
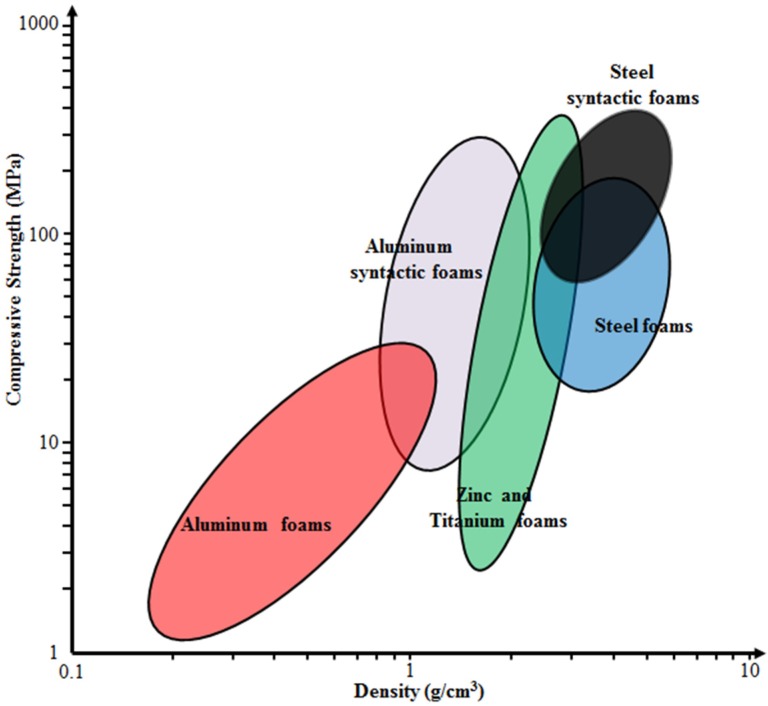
Approximate quantitative comparison of aluminum, titanium, zinc and steel conventional and SFs’ (syntactic foams) compressive strength and density, based on a data collection by Weise *et al.* [17].

A qualitative comparison of mechanical properties of many of these materials with conventional types of metal foams is shown in Figure 1, underlining that SFs typically outperform their non-syntactic counterparts in terms of both absolute and density-normalized compressive strength, whereas lowest absolute density is often achieved through other processes [17]. Generally, in return for their high density, the steel matrix SFs have higher strength than MMSFs. In addition, they represent another option for replacing conventional steel foams, which show comparable density, but typically at lower strength.

**Figure 2:**
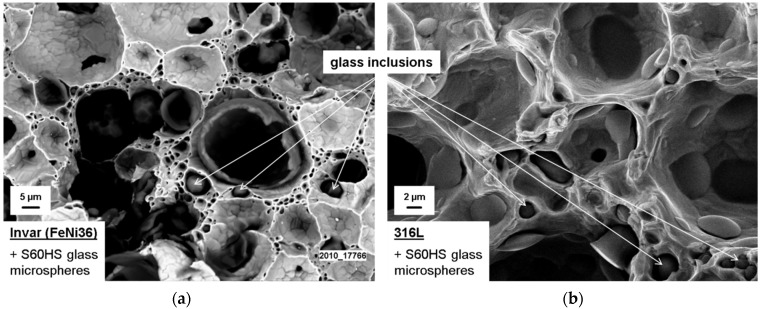
SEM images of fracture surfaces of SFs based on glass microspheres with matrices of Invar (FeNi36, (**a**)) and 316L (**b**)—glass inclusions within the matrix are visible in both cases. S60HS represents S60 hollow glass microspheres manufactured by 3M, St. Paul, MN, USA [24].

In this work, two types of SFs filled with 5 and 10 wt.% Fillite alumino-silicate cenospheres, composed, according to supplier specifications, of approximately 55–65 wt.% SiO_2_, 27–33 wt.% Al_2_O_3_ and a balance of Fe_2_O_3_, are studied [18]. Choice of these materials is based on previous experience from studies dedicated to Invar (FeNi36) matrix SFs [17,19,20,21]. Originally, the metal powder injection molding (MIM) process for production of MMSFs had been successfully developed using Fe99.7 materials as matrix [22,23]. However, observation of the tensile behavior of these foams and notably their low ductility under these conditions has led to the assumption that residual stresses brought in during the cooling step following sintering might account for this behavior, and Invar might thus be a preferable matrix material based on its coefficient of thermal expansion, which is better matched to the glass microspheres used as filler in these studies. Investigation of this effect is still ongoing, without fully conclusive results yet, even though the Invar matrix foams did in fact show the expected higher ductility under tensile load [19]. Despite this positive effect, a microstructural comparison of glass microsphere SFs with matrices of Fe99.7 (sintered at 900 °C), Invar (1000 °C) and 316L (1200 °C) revealed onset of disintegration of glass microspheres at sintering temperatures of 1000 °C, until the full loss of all spheres when sintering at 1200 °C. Figure 2 illustrates this effect on fracture surfaces of the respective materials: for Fe99.7, glass microspheres generally show spherical shape and smooth surfaces (not depicted here, see [20,23]). For Invar, the originally identical microspheres now seem less regular in shape, and satellite glass phases can be observed in between pores within the matrix (Figure 2a) For 316L, the same satellite phases dominate the image, while the inner pore surfaces only occasionally show coverage by glass, which never extends over the entire pore surface area (Figure 2b).

A similar effect is observed when comparing the experimentally measured to the theoretically expected densities (the latter based on rule of mixtures applied to matrix density, filler particle density and volume fraction of filler): the higher the sintering temperature, the greater the level to which the measured density exceeds the theoretical one. This phenomenon is usually understood as reflecting the microsphere’s lack of resistance against sinter shrinkage, which gets more pronounced the higher the sintering temperature is chosen [21].

Both effects underline the need for filler of increased thermal stability, and cenospheres comprising higher quality ceramics such as Al_2_O_3_ and SiO_2_ have potential to be useful in this regard [18,21]. Consequently, the present study scrutinizes the structural features of Invar matrix SFs filled with cenospheres rather than glass microspheres. In addition, a link to properties is established through an evaluation of compressive strength across a wide range of strain rates from quasi-static to highly dynamic.

## 2. Results

### 2.1. Density and Microstructure

**Figure 3:**
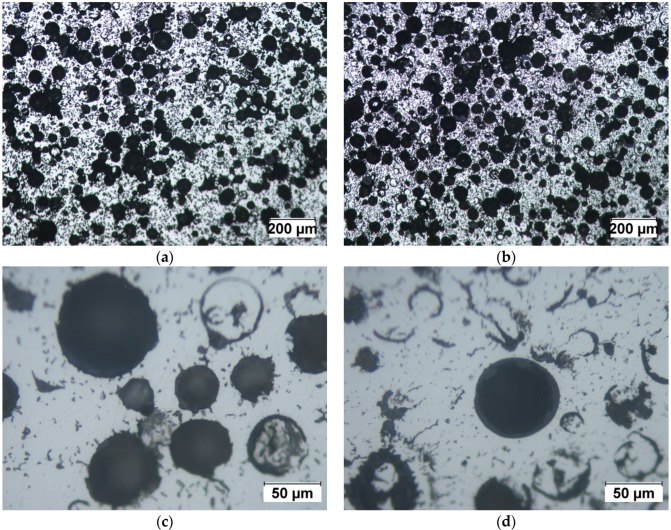
Optical micrographs of (**a**,**c**) Invar-5CS; and (**b**,**d**) Invar-10CS at different magnifications.

The measured and theoretical densities of Invar SFs are listed in Table 1. Determination of densities was done through weighing of machined samples of a well-defined volume. Two types of Invar SF filled with 5 and 10 wt.% cenospheres are denoted as Invar-5CS and Invar-10CS, respectively. For comparison, data from Luong *et al.* [20] on comparable materials containing 3M^TM^ S60HS glass microspheres, for which the average particle density is specified as 0.6 g/cm^3^ [25], are added. For these, the theoretical particle density matches the minimum value specified for the cenospheres. The theoretical densities of Invar SFs were calculated based on the density range of cenospheres (0.6–0.85 g/cm^3^) as shown in Table 3, assuming that this particle density has been retained in the process and no matrix porosity is present. The real density of both Invar-5CS and 10CS is within the expected range. Had softening of the filler been the case as with the glass microspheres, some deviation would have had to be expected already at the lower filler particle level, an effect which is clearly visible for the glass microspheres included for comparison from the preceding study [20]. Besides, further microscopic observations presented in Figure 3 do not support any such thermal degradation of the cenospheres.

**Figure 4:**
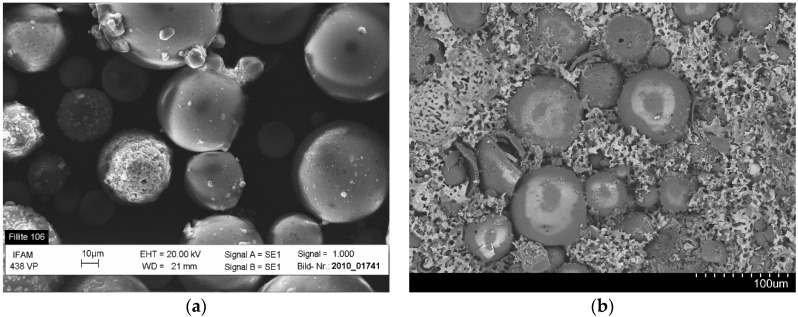
SEM images of (**a**) Fillite 106 alumino-silicate cenospheres in their virgin state before processing; and (**b**) embedded in the Invar-10CS after feedstock processing, injection molding and sintering.

Figure 3 shows the optical microstructure of Invar-5CS and 10CS at different magnifications. Both types of Invar SFs have well-dispersed cenospheres, but the filler distribution is denser for the Invar-10CS. At high magnification in optical micrographs, cenospheres that are broken and filled by the Invar matrix material during the synthesis process are observed. These appear to be more numerous in the structure of Invar-10CS. Some of the filled cenospheres on the exposed surface are due to the deformation of the matrix during polishing before optical microscopy. The density observations in Table 1 show that the fraction of broken cenospheres is small because the experimentally measured density is within the expected range. Figure 3d also shows a seemingly well-bonded interface between the Invar matrix material and the cenospheres. However, no statement about the actual bond strength is possible. Focused Ion Beam (FIB) based investigations on 316L matrices reinforced with glass microspheres in [17] show a clear lack of interface strength, and fracture surfaces of specimens with both 316L and Invar matrices indicate glass microspheres and glass inclusions within the matrix as the main failure initiation sites (see also Figure 2 in this respect). Though differences between glass microspheres and cenospheres in terms of composition suggest the possibility of a deviation in this respect, the current experimental results do not suffice to substantiate this. In order to investigate the effect of the synthesis method on cenospheres, the Invar SFs were immersed in 1.0 vol.% nitric acid for 30 min in order to lay bare the cenospheres originally enclosed in the matrix. The observation of the corrosion face by scanning electron microscope (SEM) equipment would provide the state of cenospheres in the structure of Invar SFs without any physical effects. Figure 4a shows the original cenospheres, while Figure 4b presents the surface of the syntactic foam after having been subjected to the corrosive environment. In Figure 4b, no significant geometrical deformation of the Fillite alumino-silicate cenospheres can be seen in comparison to their original state as shown in Figure 4a. This is explained by the cenosphere’s high melting temperature of 1200 °C (Table 3), which is significantly higher than the sintering temperature during the MIM synthesis procedure (1000 °C).

### 2.2. Compressive Properties

**Figure 5:**
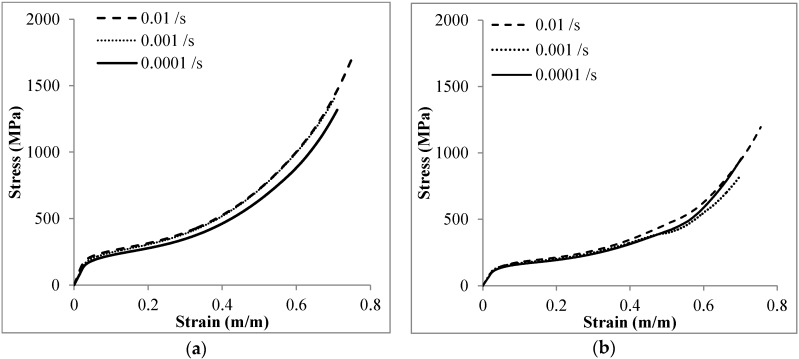
Representative of the compressive stress-strain response of: (**a**) Invar-5CS; and (**b**) Invar-10CS at strain rate of 10^−2^, 10^−3^ and 10^−4^·s^−1^.

The engineering compressive stress-strain behavior of Invar SFs at quasi-static strain rates is plotted in Figure 5. Generally the curve of the Invar SFs can be separated into three regions: elastic, plateau, and densification. A smooth yielding transition is observed between the elastic and plateau regions. The yield strength is therefore specified by the offset method, using 0.2% of plastic strain to define a proof stress-type yield limit and thus separate the elastic and plastic regions. The stress still increases gradually in the plateau region. Since this change is slow but continuous and extends from the plateau into the densification region, there is no significant point separating these two strain ranges. Thus the densification strain has been specified as the intersection of tangent lines formed by the strain range of 10%–30% in the plateau region and another of 60%–80% in the densification region. The yield strength, densification strain and energy absorption at the strain of 50% and densification strain of Invar-5CS and Invar-10CS are listed in Table 2.

**Figure 6:**
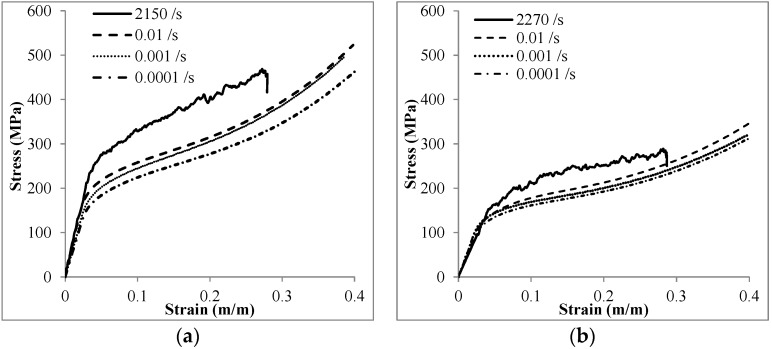
The compressive response of (**a**) Invar-5CS; and (**b**) Invar-10CS at high strain rate loading in comparison to the quasi-static loading.

In order to study the strain rate sensitivity of compressive properties, Invar SFs were tested at high strain rate loading by using a Split Hopkinson pressure bar (SHPB) set up. The details of the SHPB procedure are presented in Section 4. The strain rate sensitivity of the compressive stress-strain behavior is clearly shown in Figure 6, in which the stress level both for Invar-5CS and Invar-10CS samples shows an increasing trend with strain rate. It should be noted that the end of compression in SHPB testing may not represent specimen densification or failure as the total strain that can be obtained in SHPB for a given strain rate depends on the test instrumentation. It is also noted that the strain rate is recovered from the test results, therefore, behavior of two MMSFs at exactly the same strain rates cannot be compared.

## 3. Discussion

**Figure 7:**
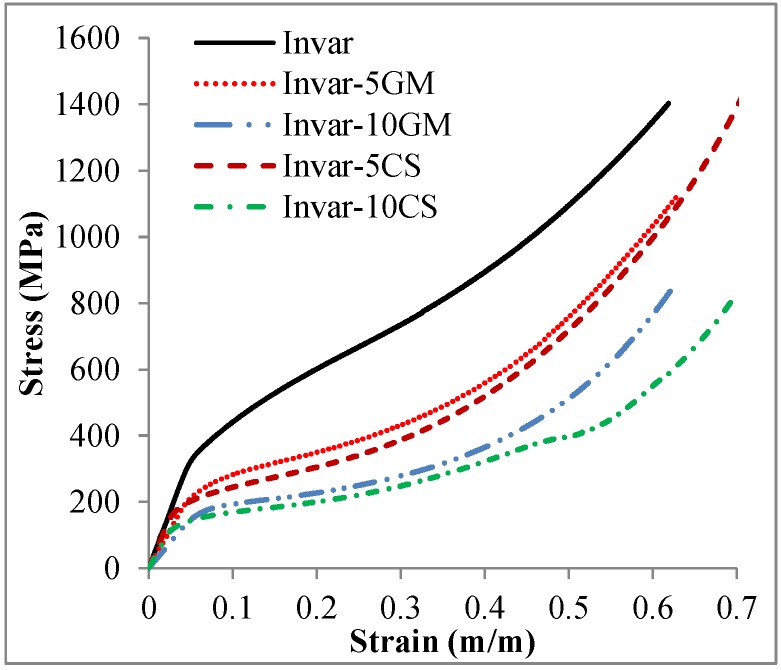
The compressive stress-strain response of Invar steel and its SFs at the strain rate of 10^−3^·s^−1^. The data of Invar and its SFs-filled glass microballoons (GM) is obtained from reference [20].

Figure 7 shows the engineering compressive stress-strain behavior of the Invar material and its SFs filled with different kinds of hollow particles at a strain rate of 10^−3^·s^−1^. Invar exhibits higher strength in comparison to its SFs at the same strain level. Additionally, the Invar SFs filled with cenospheres have lower strength than the Invar-GM SFs having comparable density [20]. In view of the expected positive influence of the cenospheres on structural features of the foams, the actual realization of which is supported by the density measurements reported in Table 1 as well as the optical micrographs depicted in Figure 3, this may seem surprising. It must however be assumed that the said structural effect is counterbalanced by the generally lower strength of the cenospheres (see Section 4, Table 3, on filler particle characteristics according to supplier specification). This lower strength can be explained by defects in the shell resulting in a generally lower quality of cenospheres, which are an industrial waste product [6]. In general, this observation is another indication of the fact that iron and steel matrix SFs would greatly benefit from higher strength and stiffness, but having at the same time thermally stable microspheres as advanced filler particles. Currently, such alternatives, for which ceramics like SiC or Al_2_O_3_ would be attractive candidates, are not available in the small size ranges matching the requirements of the MIM process, *i.e.*, at diameters roughly below 100 µm. From the available experimental data, the true potential of such optimized iron/steel matrix SFs is hard to estimate, since, in the present case, structural effects and filler particle characteristics are superimposed.

**Figure 8:**
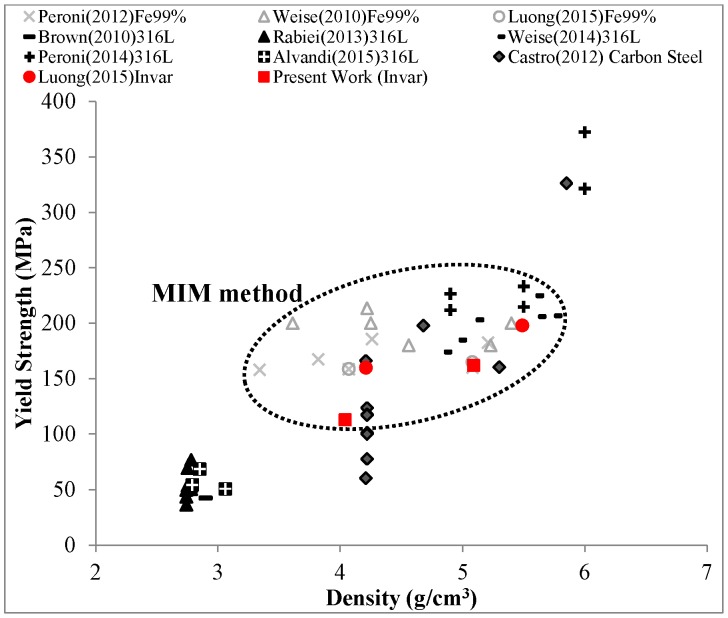
The yield strength of Invar SFs-filled cenospheres in comparison to other steel based matrix SFs. The data is generated and collected from references [13,14,17,20,21,22,28,29,30,31].

The yielding behavior of SFs is controlled by a complex concurrence of processes, including the matrix material plastic deformation and the hollow particles breakage. Other kinds of SFs such as the aluminum matrix variants reported by [6,26] show a peak stress between the elastic and plateau regions. The cracks that propagate to the matrix material from the broken cell wall of hollow particles were observed, while the stress had a significant drop [26,27]. The cracks lead to the structure collapsing under the compressive loading. The development of cracks in the matrix is due to two reasons: (1) a strong interfacial bonding between matrix and fillers [26]; (2) the insufficient fracture toughness of matrix material to plastically prevent cracks [14]. In contrast, the yielding transition between the elastic and plateau regions of Invar SFs is a smooth curve. It clearly shows that there is no significant collapse in structure of matrix material after the cenopheres fail. Due to the fact that yielding transition is a distinct segment, the yield strength specified by the offset strain of 0.2% should be used to define the end of the elastic region and the beginning of material yielding. The yield strength of Invar SFs from the present work is plotted over density in Figure 8 and contrasted with corresponding values of other steel SFs collected from references [13,14,17,20,21,22,28,29,30,31]. Generally, the yield strength increases with density, thus showing the same trend as reported earlier for other variants of MMSFs in [6,20]. According to Figure 8, Invar SFs filled with cenospheres provide lower yield strength than other steel SFs synthesized via the MIM route, but containing glass microballoons. As discussed above, this observation can be related to the lowered mechanical characteristics of cenospheres (1500–3000 psi or 10.3–20.7 MPa [18], compared to 18,000 psi or 124.1 MPa for S60HS glass microballoons [24]). However, these retain their relevance, as they can provide other options for structural applications requiring high strength over 100 MPa, which only few other MMSFs reach [6].

**Figure 9:**
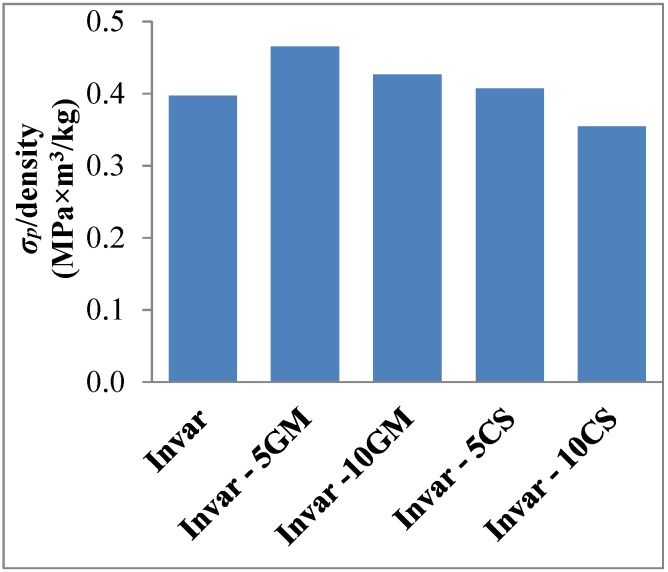
The plateau onset stress over density of Invar SFs in comparison to the yield strength over density of Invar matrix materials. The data of Invar SFs-filled glass microballoons is reported in reference [20].

In order to evaluate the compressive mechanical performance with the contribution of cenosphere strength, the plateau onset strength, which is the stress at the intersection between tangent lines of the elastic and plateau regions, was specified [32]. The plateau onset stress is considered as a point at which plastic deformation of matrix material and filler fracture happen at the same time. Its value for Invar-5CS and Invar-10CS is 207 and 143 MPa, respectively. In comparison to the yield strength (306 MPa) of the pure Invar matrix material [20], Invar-5CS and Invar-10CS have a 32% and 53% lower strength. This is no surprise result because the Invar matrix material has a solid structure and thus higher density than its SF structure. This point has previously been stressed when contrasting Invar-5GM and Invar-10GM material variants with their cenosphere-based counterparts. The reduced strength of the latter, which at first sight seems to contradict the improvement in structural aspects, is likely the reflection of the lower strength of the filler particles. The plateau onset stress over density of Invar SFs and the matrix yield strength over density are plotted in Figure 9. After eliminating the effect of density on the plateau onset stress value, the results obtained for Invar-5CS materials can be comparable to those associated with a pure Invar matrix. Figure 9 illustrates this effect of the filler on SF strength.

**Figure 10:**
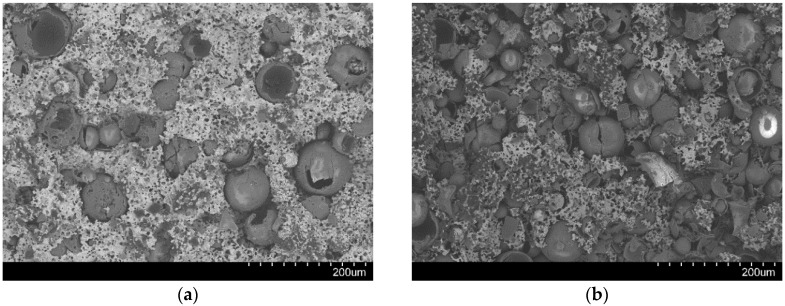
SEM images showing the cenosphere fracture in: (**a**) Invar-5CS; and (**b**) Invar-10CS at a strain of 5%.

**Figure 11:**
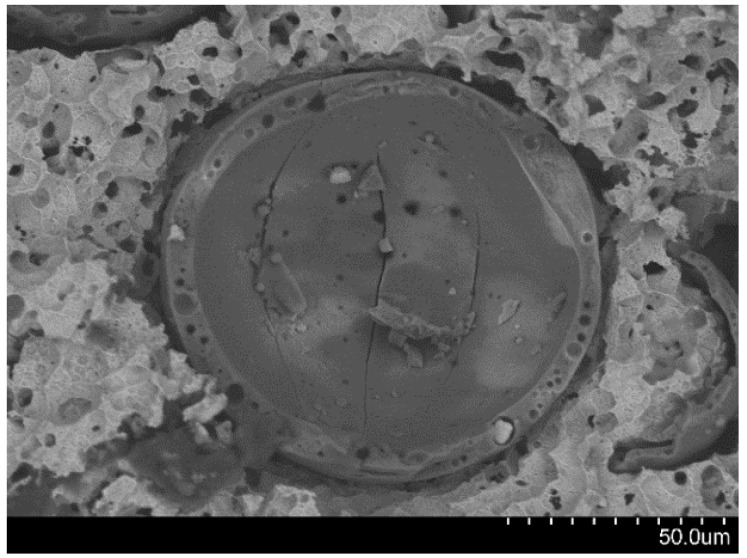
The pattern of crack development on cenospheres under quasi-static loading in the top-bottom direction.

In the plateau region, stress levels show a gradual increase, impeding the distinction between the former and the densification region. To investigate the state of cenospheres in this region, the Invar-CS specimens were tested at a strain rate of 10^−3^·s^−1^, and deformation was stopped at an early stage within the plateau region (5% strain) and the samples subsequently etched in nitric acid of 1.0 vol.% to reveal the state of the cenospheres at this point. As shown in Figure 10, most cenospheres had already failed at this early stage of the plateau region. The respective crack can be seen on the shell and has the same pattern, developing in the same direction of loading (Figure 11). Possible explanations of this effect include progressive strain hardening as a consequence of density gradients and stress localization in the porous structure. In stochastic foams like those produced in the liquid state via blowing agents, such effects have been alternatively interpreted as consecutive compression of regions of increasing density stacked in the direction of compression [33], as a result of dominating ductile failure of the matrix and ensuing strain hardening [34], or as combinations of both effects [34,35]. In the present case, due to the greater homogeneity of SFs, strain hardening may be considered the dominant effect. It is worth noting in this respect that, contrary to many infiltration-based SFs [8], in MIM-based materials, the filler particles do not form a continuous, brittle network within the matrix, but are dispersed, allowing transfer of part of the ductility of the matrix to the combination of filler and matrix. Alternatively, Peroni *et al.* [23] have suggested a mechanism for development of deformation bands in syntactic foams: the inhomgeneity of the stress distribution might lead to local initiation of failure at peak stress sites. Failure of microspheres at such sites would weaken the respective cross-section and lead to it becoming a preferred site of deformation. Similar effects have been observed computationally in polymer matrix syntactic foams, where fracture of weaker particles causes localized stress concentration, leading to other hollow particles in the vicinity breaking until the stress is sufficiently low [36]. Strain hardening and local densification would lead to strengthening of this initial deformation band and initiation of plastic deformation, first through failure of microspheres in other, now weakest, cross sections [22,23].

**Figure 12:**
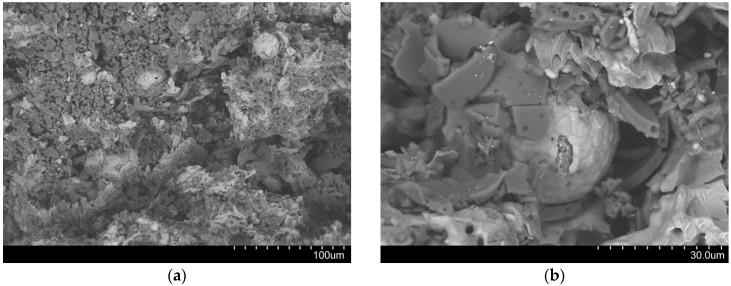
SEM images of fracture face on the Invar-10CS under quasi-static loading at magnification of (**a**) ×400; and (**b**) ×1500.

**Figure 13:**
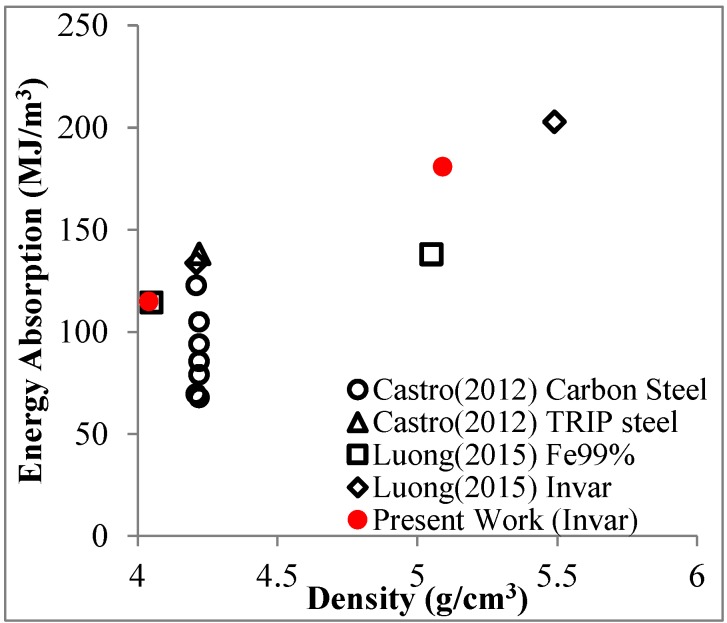
The energy absorption up to the strain of 50% of steel-based matrix SFs.

Crushing of cenosphere fragments within the pores they originally lined may come in as an additional, secondary explanation. Figure 12a shows the facture face near the border of Invar-10CS specimen after the densification point. The crushed cell walls of cenospheres are easily recognized as darker fragments, while the Invar matrix shows up in a brighter color. The cell walls were debonded from the matrix during the plateau region as shown in Figure 12b. It can be explained by debonding development following the gap at the interface between the matrix and filler that is constituted during the cooling process from the sintering temperature by the difference of their thermal expansion coefficient [28]. Since the SFs are porous structures, the energy absorption capacity of Invar SFs mainly related to the plateau region is of interest in order to judge the materials’ application potential. Figure 13 plots the energy absorption capacity of the presently studied materials at a total strain of 50% in comparison to corresponding results obtained for other types of iron/steel SFs [14,20]. The diagram obtained shows that already, in combination with cenospheres, the material shows characteristics that compare favorably with the related materials considered here. In the lower density range around 4–4.5g/cm^3^, the higher carbon content level carbon steel syntactic foams studied by Castro *et al.* [13,14] reach similar energy absorption capacity levels, though at a significantly more brittle overall behavior. Transformation induced plasticity (TRIP) steels also studied by Castro *et al.* [14] promise more characteristics in this respect and almost exactly match MIM-based Invar-CS SFs in terms of energy absorption. In these studies, however, the steel matrices are combined with higher strength ceramic microspheres [14]. As has been pointed out repeatedly above, availability of these materials as micro-scale fillers suitable for the MIM process would be expected to significantly raise the properties of our materials as well. Besides, in view of differences in part sizes (typically larger in the melt-based process suggested by Castro *et al.* [13,14]) and geometrical complexity (typically higher in the powder metallurgical MIM process), these two material synthesis variants must be seen as docile complementary rather than competing technologies.

**Figure 14:**
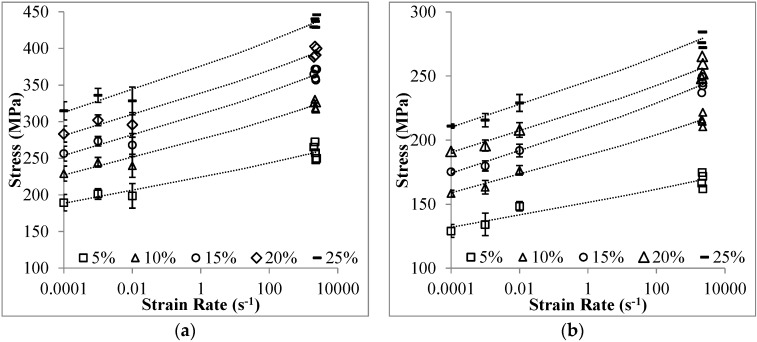
Stresses of (**a**) Invar-5CS; and (**b**) Invar-10CS at the strain from 5% to 25% plotted over strain rate.

**Figure 15:**
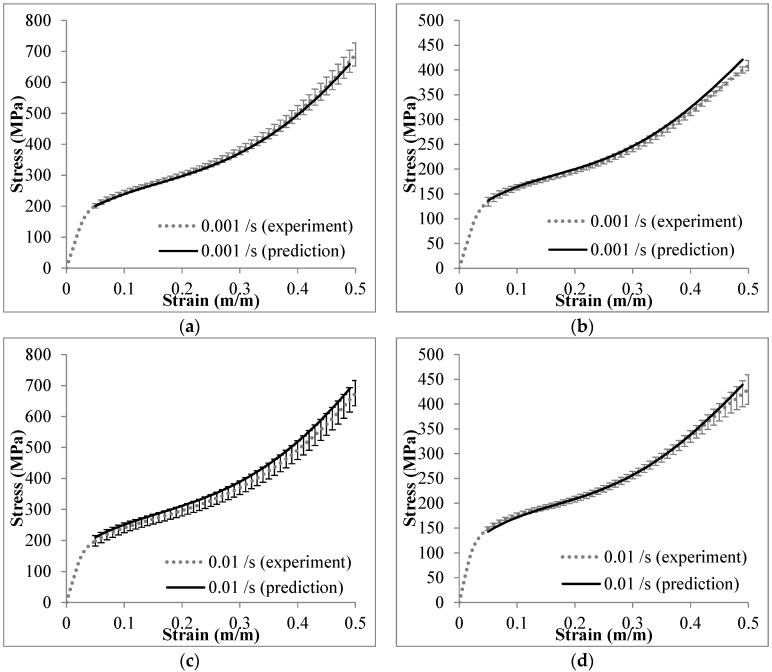

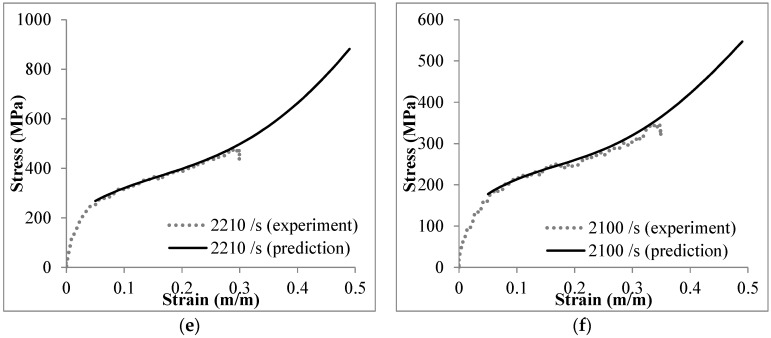
Comparison between the experimental and prediction results of: (**a**,**c**,**e**) Invar-5CS; and (**b**,**d**,**f**) Invar-10CS at different strain rates.

The strain rate sensitivity of a material at a certain strain level is expressed by [37]:

(1) $$\frac{\sigma_{1}}{\sigma_{2}}={\left(\frac{{\dot{\varepsilon }}_{1}}{{\dot{\varepsilon }}_{2}}\right)}^{m}$$

where *σ*_1_ and *σ*_2_ are stresses at identical strain values corresponding to different strain rates of ${\dot{\varepsilon }}_{1}$ and ${\dot{\varepsilon }}_{2}$, respectively; the exponent *m* represents the strain rate sensitivity. The stresses at strain from 5% to 25% are plotted over the logarithmic scale of strain rate in Figure 14. The power-law fitting curves are generated for these stresses over strain rate from 10^−4^ to 2500 s^−1^. The fitting curves are mostly inside the range of stress deviation. The strain rate sensitivity parameter *m* is the slope of the dashed lines as plotted in Figure 14. The parameter *m* is specified as 0.020 and 0.018 for Invar-5CS and Invar-10CS, respectively. Based on this formulation, the stress at an arbitrary strain rate can be predicted if the stress value at certain strain rates and the strain rate relation (*m* parameter) are known. The plateau stresses in the range of 5%–50% strain for Invar-CS SFs are constructed from the one at strain rate of 10^−4^·s^−1^, and the predictions for three individual, deviating strain rates, plotted in Figure 15. The predicted result at each quasi-static strain rate is compared to the average stress-strain curve with the standard deviation (see Figure 15a–d), and the one at high strain rate is directly compared to the experimental curve (see Figure 15e,f). Predicted stresses receive higher error markings at the beginning and end stage of the plateau region, an observation which is associated with the fact that in yielding and densification transition zones, higher complexity of deformation processes makes these less predictable. However, the error between the experimental and prediction results is reported at less than 10%.

## 4. Materials and Methods

**Figure 16:**
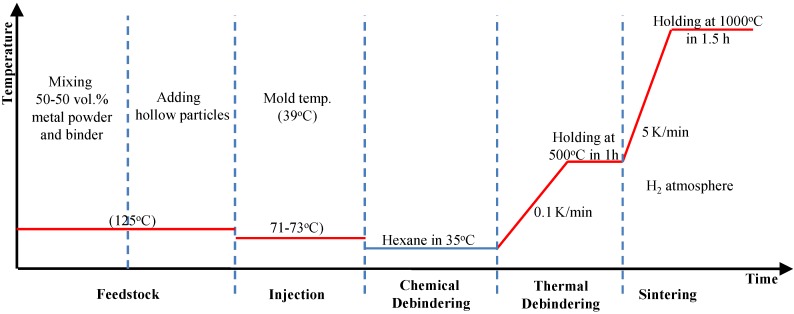
The metal powder injection molding (MIM) process of Invar SFs.

Two types of Invar SFs-filled 5 and 10 wt.% cenospheres were synthesized by the metal powder injection molding method (MIM), which has been described in detail in relation to MMSFs, e.g., by Peroni *et al.* [23] and by Weise *et al.* [19]. The Invar composition of 64 wt% Fe and 36 wt% Ni was achieved by mixing the respective elementary powders. The Omya Fillite^®^ FG (106) Alumino-silicate Cenospheres (Omya GmbH, D-50679 Köln, Germany)were used and salient properties obtained from the datasheet provided by the supplier are listed in Table 3 [18]. The MIM process is depicted in Figure 16. In the first step, the metal powders were mixed with the binder material (polymer-wax blend, 50 vol.% related to the metal-binder mixture without hollow particles) followed by cenospheres to yield the homogeneous feedstock, which is injected into the mold at a pressure of 10 bar. The temperatures of mixing and injection steps are 125 °C and 71–73 °C (mold temperature 39 °C), respectively. The binder was removed from the green part by chemical (Hexane solvent, 24h at 35 °C) and thermal steps (heating up at 0.1 K/min to 500 °C and holding at this temperature for 1 h). Finally the sample was sintered at a temperature of 1000 °C, and this temperature was ramped up at 5 K/min, with a holding time of 1.5 h in a hydrogen atmosphere. Afterwards, sintered parts where slowly cooled to room temperature within the furnace. Previous studies on Invar and 316L matrices have indicated that neither the configuration of the initial powder mixture [19] nor the duration of the final sintering step [17] have a dominant influence on the mechanical properties.

In order to study the compressive properties of Invar SFs under the quasi-static and high strain rate loading, cylindrical specimens, with a ratio of length to diameter of 1:1, were machined. The average diameters of Invar-5CS and Invar 10-CS specimens were 4.4 and 4.6 mm, respectively. An Instron 4469 machine (Intron, Norwood, MA, USA) with 50 kN load cell was used to conduct the quasi-static compression tests on 3 specimens of each type with the initial nominal strain rates of 10^−4^, 10^−3^ and 10^−2^·s^−1^. A lubricant of Dow Corning-Molykote 111 (Ellsworth Adhesives, Germantown, WI, USA) was used to minimize the friction effect between the cylindrical specimens and compression platens.

The high strain rate compression tests were conducted by using a split-Hopkinson pressure bar (SHPB) setup. The Invar SF specimen was sandwiched between the incident and transmitter bars. The incident and transmitter bars used in these tests were made of Inconel steel. The stress (σ); strain (ε); and strain rate ($\dot{\varepsilon }$) were calculated by the following equations:

(2) $$\dot{\varepsilon }\left(t\right)=\frac{2c_{b}\varepsilon_{r}\left(t\right)}{l_{0}}$$

(3) $$\sigma \left(t\right)=\frac{AE\varepsilon_{t}\left(t\right)}{A_{0}}$$

(4) $$\varepsilon \left(t\right)=\int_{0}^{\tau }\dot{\varepsilon }\left(t\right)d\tau$$

where $\varepsilon_{r}\left(t\right)$ and $\varepsilon_{t}\left(t\right)$ are the reflected and transmitted axial strain pulses, respectively, with respect to time; in addition, *A* and *E* are the cross-section area and Young’s modulus of the bar materials, respectively; *l*_0_ and *A*_0_ are the initial length and cross-section area of the specimen, respectively; *c_b_* is the sound wave velocity in the bar; and τ is a time variable used for integration. The representative strain rate for each stress-strain curve was calculated as the average value of the plateau region of the strain rate-strain graph. The details about the in-house developed SHPB configuration can be found in references [9,12,20,26,38]. The microstructure of Invar SFs before and after the mechanical experiments was observed by using the Hitachi S-3400N scanning electron microscope (SEM, Hitachi, Tokyo, Japan) and a Nikon EPIPHOT 200 microscope fitted with a Nikon DS-Fi1 digital camera (Nikon, Shizuoka, Japan).

## 5. Conclusions

In the present work, Invar-cenosphere SFs were investigated in terms of their compressive properties in the strain rate range from 10^−4^ to 2500 s^−1^. The results show that they are sensitive to strain rate in this range. Their rate-dependent compressive characteristics are experimentally determined by a model that describes stress at given strain levels within the plateau range, allowing the derivation of the respective stress-strain curves for arbitrary rate values within the experimentally covered range. With regard to processing of the materials and the resulting microstructures, this study has confirmed the notion that the higher thermal stability of cenospheres makes them a better choice for powder metallurgy production of Invar matrix SFs than glass microspheres in terms of structural characteristics. Cenospheres clearly survive the synthesis process, while glass microspheres tend to suffer based on their softening point of 600 °C, 400 °C below the sintering temperature employed to produce Invar matrix SFs. At the same time, however, the generally lower mechanical strength of the cenospheres compared to the thermally less stable glass microspheres finds its expression in the mechanical properties of the respective SFs. In this sense, the current study underlines the notion that for optimum performance of metal- and specifically steel-based SFs, higher strength, thermally stable microspheres are mandatory. Candidate materials could be SiC or Al_2_O_3_. At present, however, these are only available as larger size hollow spheres, which makes them unsuitable for application in the metal powder injection molding or MIM process studied here. For this reason, we strongly advocate the development of micro-scale (less than 100 µm in diameter) ceramic microspheres.

## Figures and Tables

**Table 1:** The measured and theoretical densities of Invar SF with cenospheres (CS) and glass microspheres (GM) as filler.

Sample	Measured Density (g/cm^3^)	Theoretical Density (g/cm^3^)	
		Min	Max
Invar-5CS	5.09	4.99	5.68
Invar-10CS	4.04	3.60	4.37
Invar-5GM [20]	5.49	4.99	
Invar-10GM [20]	4.21	3.60	

**Table 2:** The yield strength, densification strain and energy absorption at 50% strain and densification point of Invar SFs under quasi–static loading.

Sample	Strain Rate (s^−1^)	Yield Strength (MPa)	Densification Strain (%)	Energy Absorption at 50% Strain (MJ/m^3^)	Energy Absorption at Densification Strain (MJ/m^3^)
Invar-5CS	10^−4^	152 ± 6.67	55 ± 2	170 ± 7.2	204 ± 24.6
	10^−3^	162 ± 3.85	53 ± 3	181 ± 6.8	200 ± 17.9
	10^−2^	159 ± 11.93	52 ± 3	177 ± 11.2	194 ± 28.4
Invar-10CS	10^−4^	109 ± 1.26	51 ± 2	113 ± 1.2	118 ± 7.5
	10^−3^	113 ± 3.60	51 ± 2	115 ± 2.7	119 ± 8.2
	10^−2^	113 ± 5.58	53 ± 0	123 ± 3.6	136 ± 3.8

**Table 3:** The datasheet of Omya Fillite^®^ FG (106) alumino-silicate cenospheres [18].

Properties	Values
bulk density (g/cm^3^)	0.350–0.450
particle density (g/cm^3^)	0.600–0.850
particle size range (μm)	5.0–106
melting point (°C)	1200–1350
crushing strength (psi)	1500–3000
component elements of shell	Al_2_O_3_ (27%–33%), SiO_2_ (55%–65%), Fe_2_O_3_ (6% maximum)
